# First report of chemical composition and cytotoxicity evaluation of *Foraminispora rugosa* basidiomata from Brazil

**DOI:** 10.1186/s40529-022-00363-8

**Published:** 2022-11-26

**Authors:** V. O. Garcia, M. Fronza, R. Von Borowski, G. Alves-Silva, A. R. Zimmer, T. Ruaro, S. C. B. Gnoatto, A. Dallegrave, R. M. B. Silveira

**Affiliations:** 1 Institute of Biosciences, Federal University of Rio Grande do Sul, Av. Bento Gonçalves 9500, Porto Alegre, Rio Grande do Sul 91501-970 Brazil; 2 University of Vila Velha, Avenida Comissário José Dantas de Melo, 21 - Boa Vista II, Espírito Santo, 29102-920 Brazil; 3 Faculty of Pharmaceutical Sciences, Federal University of Rio Grande do Sul, Av. Ipiranga 2752, Porto Alegre, Rio Grande do Sul 90610-000 Brazil; 4 Institute of Chemistry, Federal University of Rio Grande do Sul, Av. Bento Gonçalves 9500, Porto Alegre, Rio Grande do Sul 91501-970 Brazil; 5 Av. Roraima, 1000. Prédio 21, Sala 5231, Santa Maria, 97105-900 Brazil

**Keywords:** Cytotoxicity, *Foraminispora rugosa*, *Ganodermataceae*, Oxylipins, Terpenoids, Tandem mass spectra

## Abstract

**Background:**

*Foraminispora rugosa* is a species reported from Brazil, Venezuela, French Guiana, Costa Rica and Cuba. It is a basidiomycete in the *Ganodermataceae* family. In this study, both chemical composition and cytotoxicity of the ethanolic extract of *F. rugosa* were investigated for the first time.

**Results:**

Phylogenetic analysis confirmed the identification of the specimens, and the results of cytotoxicity assays showed that at concentrations of 7.8–500.0 µg/mL the ethanolic extract displayed weak cytotoxicity against the tested cell lines. Five oxylipins were identified by ultra high performance liquid chromatography coupled with quadrupole time-of-flight and mass spectrometry (UHPLC-QTOF–MS).

**Conclusions:**

This study provides new insights into the current knowledge of bioactive compounds produced by macrofungi, and provides data for future biological assays with relative selectivity and safety.

**Supplementary Information:**

The online version contains supplementary material available at 10.1186/s40529-022-00363-8.

## Background

Among medicinal fungi, those belonging to the *Ganodermataceae* family are the most widely studied in the world, principally the genera *Ganoderma* and *Amauroderma*. *Ganoderma lingzhi* (misidentified as ‘*G. lucidum*’) (Ahmad et al. 2021) has been used in traditional Chinese Medicine for more than 2000 years and it is an essential part of Chinese traditional culture, boosting scientific research in the area of health and well-being, and moving billions of dollars every year (Dai et al. 2017). In addition, the genus *Amauroderma* contains approximately 30 species, most of them widespread in tropical areas (Kirk et al. 2008). Examples of bioactive compounds isolated from *Amauroderma* are: amaurocine, a protein with anti-*Trichomonas vaginalis* activity (Duarte et al. 2016); ergostherol or the pro-vitamin D2 (Li et al. 2015); compounds with anti-acetylcholinesterase activity as amauroamoienin (Zhang et al. 2013); jacareubin, a xanthone with cytotoxic activity against tumor cell lines of human epidermoid carcinoma and cervical carcinoma (Tang et al. 2015; Kaennakam et al. 2015).

Costa-Rezende et al. (2017) found that some neotropical *Amauroderma* species do not fit within the phylogenetic delimitation of *Amauroderma *sensu stricto. The specimens once called *Amauroderma sprucei* were relatively distant from the *Amauroderma s. str*. clade. Through ultramicroscopic studies, the researchers proved that these specimens form a new taxon, the genus *Foraminispora.* This genus was characterized by presenting differentiated ultramicroscopic structures, namely hollow pillars that join the endospore to the exospore. No reports are found in the literature for the chemical or biological evaluation of *Foraminispora rugosa* (Berk.) Costa-Rezende, Drechsler-Santos & Robledo*,* a Brazilian species. Therefore, due to the important role that fungi within *Ganodermataceae* play in the pharmacological field, and also due to the lack of chemical knowledge about Brazilian funga (Kuhar et al. 2018), the ethanolic extracts of *F. rugosa* were evaluated for the first time, by ultra-high-performance liquid chromatography coupled to mass spectrometry (UHPLC-QTOF–MS) and biological assays.

## Methods

### Fungal sampling and morphological analysis

Two *F. rugosa* specimen samples, referred to as VOG127 (n° voucher: ICN 200398) and GAS1084 (n° voucher: ICN 200399) from here forward, were collected in February 2017 in Campo Mourão (Lago Azul State Park, 24° 6′ 3″ S 52° 19′ 2″ W), Paraná, Brazil. Both specimens were dried and deposited in the herbarium at the Institute of Biosciences, at the Federal University of Rio Grande do Sul, Porto Alegre, Brazil. Morphological analysis was conducted according to Costa-Rezende et al. (2017).

### DNA isolation and amplification

Genomic DNA was extracted from dried specimens according to Góes-Neto et al. (2005). The primer pairs ITS8-F/ITS6-R and LR0R/LR7 were used to amplify the nuclear ribosomal internal transcribed spacers (ITS; ITS1-5.8S-ITS2) and the nuclear ribosomal large subunit (LSU, 28S) regions according to Dentinger et al. (2010) and Vilgalys and Hester (1990), respectively. Polymerase chain reaction (PCR) was performed with a total volume of 25 μL containing 1 unit Taq DNA polymerase, 0.25 μL of 10× Taq polymerase reaction buffer (Applied Biological Material, Vancouver, Canada), 2.5 μL of bovine serum albumin solution (Sigma-Aldrich, St. Louis, Missouri), 0.25 μM of dNTP mix, 0.6 μL of each of the two 10 μM primers, and 1–2 μL of total DNA. PCR amplification of ITS was performed with 5 cycles of initial denaturation (95 °C for 30 s, 60 °C for 30 s, 72 °C for 1 min), followed by extension (30 s at 95 °C, followed by 30 s at 55 °C, 1 min at 72 °C) repeated for 25 cycles with a final extension of 10 min at 72 °C. PCR amplification of 28S followed methods by Vilgalys and Hester (1990). Purification and DNA sequencing were performed by Macrogen (Geumcheon-gu, Korea) and Fiocruz Belo Horizonte Plataforma PDTIS (Minas Gerais, Brazil). For ITS, the primers were the same as for amplification, and for 28S, we used LR0R and LR5.

### Alignment and phylogenetic reconstructions

The sequences were blasted in GenBank with Blastn searches. Based on the blast analysis and related literature, additional related sequences were assembled. ITS and LSU sequence data sets were generated. Both data sets were separately aligned using MAFFT v.7 (http://mafft.cbrc.jp/alignment/software) and manually adjusted using MEGA 7 to allow maximum alignment and maximum sequence similarity (Katoh and Standley 2013; Kumar et al. 2016). The data sets were then combined, and in the subsequent analyses were subdivided into four data partitions: ITS1, 5.8S, ITS2 and 28S. Phylogenetic analyses were carried out with combined ITS + 28S sequence data sets. In total, 54 specimens were observed (two outgroup species). The 28S matrix was built up to domain D3 (LR5 primer). *Perenniporia medulla-panis* was designated as an outgroup based on previous studies (Costa-Rezende et al. 2017). All materials and sequences used in this study are listed in Additional file 1.

All phylogenetic analyses were performed online using the CIPRES Science Gateway. We analyzed the data sets separately using maximum likelihood and Bayesian inference (BI). Maximum likelihood (ML) analysis was carried out in RAxML 8.2.9 (Stamatakis 2014). The partition file was provided to force RAxML software to search for a separate evolution model for each dataset. To access the reliability of the nodes, rapid bootstrapping replicates were computed under the same model, allowing the program to halt bootstrapping automatically by extended majority rule (MRE)-based bootstopping criterion (Pattengale et al. 2010). Bootstrap (BS) values above 80 were considered significant (high support), and above 70 were considered moderately supported. BI was performed in MrBayes 3.2.6 (Ronquist et al. 2012), and evolutionary models for BI were estimated using the Akaike information criterion (AIC) for each partition, as implemented in jModelTest2 (Darriba et al. 2012). The best-fit models for each partition were implemented as partition-specific models within partitioned mixed-model analyses (TIM1ef + G for ITS1, JC for 5.8S, TPM3 + G for ITS2 and GTR + I + G for 28S). The Bayesian analyses were conducted with two independent runs, each with four simultaneous chains for 5 × 10^7^ generations, sampling trees at every 100th generation. The convergence diagnostic was calculated every 10^4^ generation, and its critical value was set to stop the analysis automatically when the standard deviation of the split frequencies reached the value defined by the stopval command (stoprule = yes, stopval = 0.01). In all analyses, the first 25% of trees from each run were discarded as burn-in. Resulting trees from the two independent runs were then pooled to produce one, 50% majority-rule consensus tree, and Bayesian posterior probabilities (BPPs) were generated for the resulting tree. A BPP value above 0.99 was considered significant (high support), and above 0.95 was considered moderately supported.

### Preparation of *F. rugosa* ethanolic extract and fractions

Only the VOG127 specimen was used to prepare the ethanolic extract. First, 57.62 g of the material was cut into small pieces and ground in a blender. The powder was macerated with ethanol (95%) for 3 days (ratio 1:10) with solvent exchange every 24 h. After filtration by sintered funnel (G3), the solvent was removed in a vacuum with a rotavaporator (Büchi Labortechnik AG, Flawil, Switzerland). The dried extracts were stored in flasks and quantified. The ethanolic extract from VOG127 was subjected to fractionation using column chromatography (Silica Gel 60), employing gradient elution (10% increments) with ciclohexane, ciclohexane/dichloromethane, dichloromethane, dichloromethane/methanol and finally, with methanol.

### UHPLC-QTOF–MS analysis

The analysis of the five samples was carried out using a UHPLC-QTOF–MS system. The UHPLC (Shimadzu-Nexera x2) was equipped with a Shim-pack XR-ODS III column (2.0 × 50 mm, 1.6 μm) from Shimadzu with the thermostat set at 35 °C and coupled to the UHPLC-QTOF–MS mass analyzer (Impact II, Bruker Daltonics). The UHPLC-QTOF–MS system was equipped with an electrospray ionization (ESI) source, operating in negative ionization mode. Within the adopted elution gradient mode, the mobile phase consisted of A: acetonitrile (0.1% formic acid) and B: aqueous phase (0.1% formic acid). The elution gradient started at 10% of A for 2 min, then was increased to 95% in the next 10 min, and kept for 3 min. Then 95% A linearly decreased to 10% in 2 min, and was kept for 5 min. The flow rate was 0.35 mL min^−1^ and the injection volume was 10 μL. The operation parameters of ESI were the following: capillary voltage 2500 V; end plate offset, 500 V; nebulizer pressure, 3 bar (N_2_); drying gas, 9 L min^−1^ (N_2_); and drying temperature, 190 °C. The UHPLC-QTOF–MS system operated in broadband collision-induced dissociation (bbCID) acquisition mode and recorded spectra over the range m/z 55 − 1000 with a scan rate of 2 Hz. This mode provides MS and MS/MS spectra at the same time, working at two different collision energies; at low collision energy (10 eV), MS spectra were acquired. At high collision energy (20 eV), no isolation took place at the quadrupole, and the ions from the preselected mass range were fragmented at the collision cell. A UHPLC-QTOF–MS external calibration was performed before each injection with a sodium formate solution. Data treatment was processed with Data Analysis 4.2 Software (Bruker Daltonics). Besides, the accurate mass measurement (error < 5 ppm).

Before data processing, an in-house formula database was established including the compound name, molecular formula, chemical structure, accurate mass, and related product ions of the compounds in the VOG127 ethanolic extract by searching from databases such as PubChem Compound (https://www.sciencedirect.com/), RIKEN MSnspectral database for phytochemicals—ReSpect (http://spectra.psc.riken.jp/), Human Metabolome Database (www.hmdb.ca) and Mass Bank of North America (https://mona.fiehnlab.ucdavis.edu/).

### In vitro cytotoxicity

The MV-3 (human melanoma), MIAPaCa-2 (human pancreatic carcinoma), SH-Sy5y (human neuroblastoma), astroglial (C-6) cell, HEp-G2 (human hepatoblastoma), L929 (mammalian fibroblast) and Vero (African green monkey kidney epithelial cells) cell lines were used for cytotoxicity screening. Cells were cultured in Dulbecco's modified Eagle medium (DMEM) supplemented with 10% fetal bovine serum (FBS), 100 IU/mL penicillin and 100 µg/mL streptomycin, at 37 °C in a humidified atmosphere containing 5% CO_2_. Cell viability was determined by the tetrazolium salt method using MTT (Denizot and Lang 1986). Briefly, cells were seeded in 96-well flat-bottom microplates at a density of approximately 12 × 10^3^ cells/well in 150 µL of DMEM. After cell attachment, serial dilutions of extract (7.8–500.0 µg/mL) in culture medium were prepared and cells were incubated for 24 h and 48 h. Doxorubicin was used as the positive control. The control group cells were treated with higher concentrations of DMSO (0.5%). After incubation, 100 µl of 3-(4,5-dimethylthiazol-2-yl)-2,5-diphenyl tetrazolium bromide (MTT) (1 mg/ml in PBS:Medium (1:1)) was added per well, and the plate was incubated for 2 h to allow the reaction of MTT by cellular mitochondrial dehydrogenases. Excess MTT was aspirated and the formazan crystals formed were dissolved with 100 µl of dimethyl sulfoxide (DMSO). Absorbance of purple formazan, proportional to the number of viable cells, was measured at 595 nm using a microplate reader (Molecular Devises, Spectra Max 190, USA). Experiments were carried out at least in triplicate.

### Statistical analysis

Statistical analyses were performed using GraphPad software (San Diego, CA, 176 USA). Data were expressed as the mean ± standard deviation (SD). Statistical variations were determined using a one- or two-way analysis of variance (ANOVA) when appropriate. Values were considered significant when *p* < 0.05.

## Results

### Morphological analysis

**Fig. 1:**
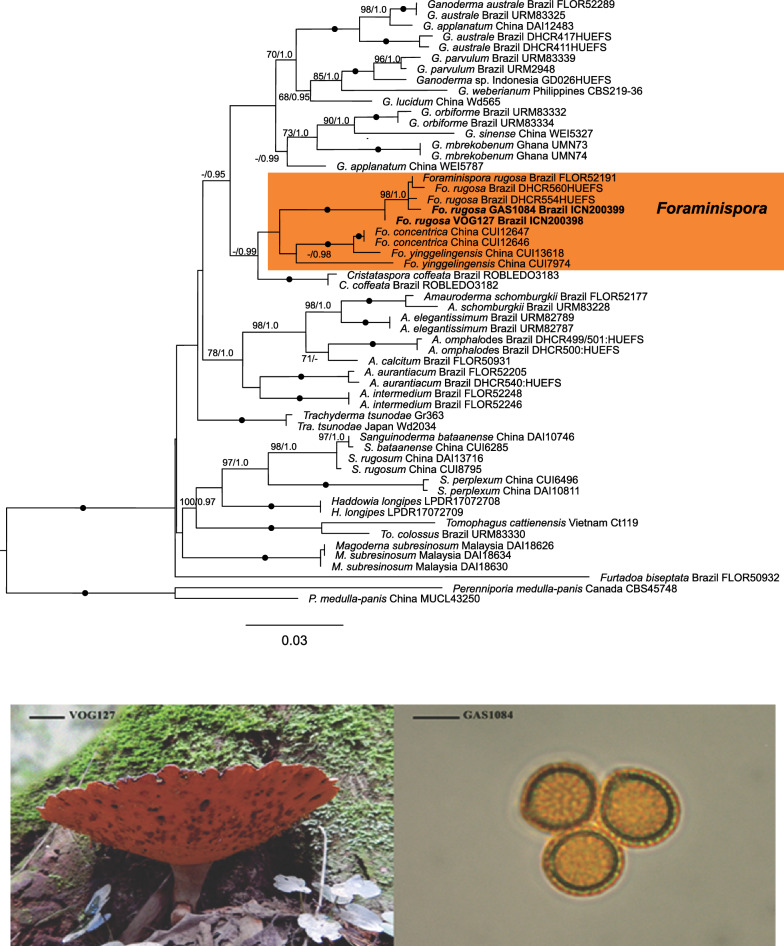
The sequences of ITS and 28S of the samples VOG127 and GAS1084

*Foraminispora rugosa* is found growing on the ground or on decayed angiosperm wood in South and Central America (Decock and Herrera-Figueroa 2006). This species is characterized by having a vivid orange hymenophore, with whitish context and the pileipellis as a short trichoderm. This basidiomycete belongs to *Ganodermatacae* for having double-walled basidiospores, with the inner layer ornamented (Fig. 1). The dimensions of the basidiospores of studied specimens were compatible with the description in the literature. Both were confirmed micromorphologically as *F. rugosa*. The sample VOG127 has subglobose basidiospores [(8.5−)8.6–10(−10.3) X (7−)7.8–9(−9.7), n = 40. Q = 1.22–1.06, Qm = 1.10, n = 40] and (5–7(-5.60)) pores/mm.

### Phylogenetic analyses

ITS BLAST queries of samples indicate the highest matches with species of *F. rugosa* with 99% similarity, and 28S BLAST showed 98% similarity. To confirm the results found in the BLAST, the sequences from the *Ganodermataceae* family available in Genbank were used. In this study, 28 species and 9 genera were used, namely: *Ganoderma*, *Foraminispora Amauroderma*, *Trachyderma, Sanguinoderma, Haddowia, Magoderna*, *Furtadoa*, and *Tomophagus*. The new ITS and LSU sequences were generated in this study for VOG127 and GAS1084. Also, the concatenated analysis of the ITS + 28S dataset was carried out and included 56 samples. The final aligned matrix of the ITS1-5.8S-ITS2 (ITS) region was 551 bp long and the 28S region, 784 bp long. The final aligned matrix of the combined dataset was 1335 bp long. The multiloci dataset recovered nine main clades. Specimens VOG127 and GAS1084 were placed as highly supported within the clade of *F*. *rugosa*. The new sequences have been deposited in GenBank (MN985326 and MN985327 for ITS; MN985507 and MN985508 for 28S). The *F. rugosa* clade was recovered in a single, well-supported lineage (100% BS,1.0 BPP) (Fig. 1) with *Ganoderma* as its sister group (–, 0.95 BPP) together with *C. coffeata* (-,0.99 BPP). *Amauroderma s.s.* (78% BS, 1.0BPP), *Trachyderma* (100% BS,1.0 BPP), *Sanguinoderma* (97% BS,1.0 BPP), *Haddowia* (100% BS,1.0 BPP), *Tomophagus* (100% BS,1.0 BPP), *Magoderna* (100% BS,1.0 BPP) and *Furtadoa* (no support value) were recovered at the genus level.

Figure 1 shows the ITS and 28S sequences of the samples VOG127 and GAS1084 (both in bold) among sequences of *F. rugosa* in the orange rectangle. The resulting topology is from maximum likelihood. The nodes with black dots indicate strong or full support (BS = 99 to 100 and PP = 0.99 to 1.0). Bottom left: the VOG127 specimen with its orange hymenophore. Scale bars: 2 cm. Bottom right: the general view of subglobose and ornate basidiospores of *F. rugosa* (GAS1084) in optical microscopy (1000 x, Melzer reagent). Scale bars: 10 µm.

### Chemical analysis

#### UPLC-QTOF-MS analysis

The solvent-free sample was weighed and yielded 1.73 g (3.01%). Then, thirty two fractions were obtained to submit the ethanolic extract to a chromatography column using solvents of increasing polarity (cyclohexane:dichloromethane:methanol). The similar fractions were collected together (according to color, polarity and number of spots) and concentrated using reduced pressure. After, five subfractions were submitted to mass analysis in UHPLC-QTOF–MS. The analysis of subfractions showed peaks in negative ionization mode. The identification of compounds was achieved by matching pseudo-molecular ion mass, values and MS/MS fragmentation patterns with online or published data of isolated compounds from the *Ganodermatacae* fungi family. Five compounds were initially identified, but many others remain unknown. All identified compounds are listed in Table 1.

**Table 1:** Characterization of compounds of ethanolic extract from *F. rugosa* by UPLC-QTof-MS^a^

N^o^/Subfraction	Tentative identification	Molecular formula	m/z (measured experimental/theoretical)	Fragment ions (m/z)	Err (ppm)	rdb	N°
8.2 (1–8)	(13-HODE) or 12(13)-EpOME	C_18_H_32_O_3_	295.2281/295.2279	295.2281; **277.2168**; 195.1386	0.8	3.5	**1**
8.3 (1–8)	13-HOtre	C_18_H_30_O_3_	293.2124/293.222	**293.2124**; 277.2167; 195.1393	0.6	4.5	**2**
8.4 (1–8)	9-KODE	C_18_H_30_O_3_	293.2122/293.2122	293.2122; 249.2216; **185.1185**	0.0	4.5	**3**
5.6 (9–10)	(9, 12, 13-TODEA	C_18_H_34_O_5_	329.2333/329.2333	**329.2333**; 293.2131; **211.1338**; 171.1018; 139.1131	0.2	2.5	**4**
5.7 (9–10)	9,10,13 TriHOME	C_18_H_34_O_5_	329.2325/329.2333	**329.2325**; 293.2108; 211.1337; **171.1022**; 139.1125	2.7	2.5	**5**

^a^Compounds are listed by order of elution; err: deviation between measured mass and theoretical mass of the respective compound in [ppm]; rdb: number of rings and double bonds in the formula. In bold: peak at 100% intensity

The most well known constituents of this family belong to the terpenoids class (Dasgupta and Acharya 2019). Thus, the starting point was to identify molecules with C15, C20 and C30. However, the fragments of the first fractions were very evident from molecules derived from fatty acids. Although these oxylipins are comprised of pairs of isomers, their structures were identified by elucidating the positions of the key hydroxyl and olefinic groups from MS/MS fragment analysis. The results demonstrate the presence of oxylipins with 18 carbon atoms (compounds **1**, **2**, **3**, **4** and **5**).

The compounds **4** and **5** are isomers with the elemental composition C_18_H_34_O_5_. In the MS/MS spectra, the main fragment m/z 211.1338 [M–H]^−^ indicates the presence of hydroxyl groups at positions 12 and 13 (**4**), and the fragment 171.1022 [M–H]^−^ of compound **5** indicates the presence of hydroxyl groups at positions 9 and 10. The compounds **4** and **5** were identified to be 9,12,13-trihydroxy-10-octadecenoic acid (9,12,13-TODEA) and 9,10,13-trihydroxy-11-octadecenoic acid (9,10,13-TriHOME), respectively (Ludovici et al. 2014; Zhao et al. 2019).

Abundant ions at m/z 293.2124 [M–H]^−^ was indicative of C_18_H_29_O_3_^−^; the peak at m/z 277.2167 indicated the loss of H_2_O and the peak 195.1393 [M–H]^−^ is a characteristic fragment from scission between C12 and C13, identical to 13-OH-9Z,11E,15Z-octadecatrienoic acid (13-HOtre, **2**) (Dufour and Loonis 2005; Ludovici et al. 2014; Trapp et al. 2015). The metabolite **3** has a main ion fragment at m/z 293.2122 and the most dominant ion at m/z 185.1185 [M–H]^–^_,_ thus, **3** was identified as (10E,12Z)-9-oxooctadeca-10,12-dienoic acid (9-KODE) (Levinson et al. 2013; Ludovici et al. 2014; Zhao et al. 2019). The signals detected at m/z 295.2291 (**1**), 277.2168 [M–H–H_2_O]^–^ and 195.1386 were attributed to C_18_H_31_O_3_ [M–H]^–^; according to the literature the compounds can be coriolic acid (3-HODE) or vernolic acid (12(13)EpOME) (Ludovici et al. 2014; Zhao et al. 2019).

#### Cytotoxicity assay

In the cytotoxicity assay, higher IC_50_ values correspond to lower cytotoxic activity. The VOG127 specimen had IC_50_ values from 119.5 μg/ml (lower dose) to 216.7 μg/ml. The cytotoxicity of the ethanolic extracts of *F. rugosa* was investigated using an MTT assay on two non-transformed cell lines and four mammalian cancer cell lines. Table 2 indicates that the VOG127 ethanolic extract had a weak toxicity for all cell lines tested when compared to doxirubicin. The extract inhibited pancreatic cancer cells (MIAPaCa-2), melanoma cells (MV-3) and L929 (non-transformed fibroblasts cells) growth at very similar concentrations (154.9 μg/mL, 151.3 μg/mL and 153.6 μg/mL, respectively). In regards to neuroblastoma (SH-Sy5y) and astroglial (C-6) cells, the inhibition caused by the ethanolic extract of the VOG127 sample was poor, while for hepatoblastoma (HEp-G2) cells and Vero cells (renal epithelial cells), the extract from VOG127 showed the strongest inhibitive activity (138.5 μg/mL and 119.5 μg/mL respectively).

**Table 2:** Cytotoxicity of the ethanolic extract of the *F. rugosa* VOG 127 sample using the MTT method

Cell lines	IC_50_*(µg/mL) values	
	VOG 127	Doxorrubicin
MV3	151.3 ± 19.3	3.83 ± 1.5
MIAPaCa-2	154.9 ± 9.1	23.53 ± 2.3
SH-Sy5y	216.7 (n = 1)	5 .00 ± 0.0^#^
C6	213.6 ± 11.8	1.31 ± 0.4^##^
HepG2	138.5 ± 3.2	1.5 ± 0.6
L929	153.6 ± 7.6	7.83 ± 0.8
Vero	119.5 ± 2.2	82.4 ± 8.7

*The activity was shown as the IC_50_ value, which was the concentration of the tested ethanolic extract (µg/mL) that decreased the number of viable cells by 50%. Results are expressed as the mean and standard error values of triplicate data points. Cell lines: MV3: human melanoma; MIAPaCa-2: human pancreas carcinoma; SH-Sy5y: human neuroblastoma; C-6: astroglial C6 cells; HEp-G2: human hepatoblastoma; L929: non-transformed mammalian fibroblast; Vero: mammalian kidney epithelial. Literature data: ^#^Namkaew et al. (2018). ^##^Zhao et al. (2016)

## Discussion

Here, we corroborate the results of taxonomic analysis found by Costa-Rezende et al. (2017). These authors observed that the columns of the endospore were hollow via Scanning Electron Microscopic analysis of the basidiospores of *F. rugosa*. Later, in 2020, other researchers found the same feature in other fungi in the Paleotropics (Sun et al. 2020). Since the VOG127 and GAS1084 samples were not evaluated through ultrastructural analysis, it was necessary to perform molecular analysis of the specimens in order to confirm their identity. Our specimen of *F. rugosa* grouped together with the specimens used by Costa-Rezende et al. (2017) and Sun et al. (2020). This categorically proved the identification of the specimens in this study by both Maximum likelihood and Bayesian data analysis.

It is known that over 90% of fungal diversity on Earth remains undiscovered (Tripp et al. 2017). Consequently, very little is known about fungal metabolites (Kuhar et al. 2018). On the contrary, the genus *Ganoderma* alone has recorded more than 400 isolated metabolites (Baby et al. 2015), unlike the other genera of *Ganodermataceae*.

In an attempt to identify the compounds of *F. rugosa*, an MS library of scientific literature through 2019 was used as a comparison for the detected fragment ions. No important ions were observed below m/z 180 or above m/z 600 on subfractions of *F. rugosa* under the analytical conditions used in this study.

Oxygenated derivatives of polyunsaturated fatty acids, the oxylipins, were found. Oxylipins are present in organisms from all kingdoms of nature and are a common means of communication among plants, animals, and fungi to control development and alter host-microbe interactions (Pohl and Kock 2014).

Oxylipins can be produced by an enzymatic or non-enzymatic route. In fungi, the enzymatic route of oxylipins acts primarily on oleic, linoleic and alpha-linolenic acids (Beccaccioli et al. 2019). The 8-hydroxylinoleic acid (8-HODE) is known to stimulate the sexual cycle of *Aspergillus nidulans* (Gessler et al. 2017) and the (5S,8R)-dihydroxy-octadecadienoic acid (5.8-diHODE) acts in the differentiation process of *A. fumigatus* and *A. flavus*. This oxylipin causes lateral branching in *Aspergillus* hyphae and appressorium formation in *Magnaporthe grisea* (Niu et al. 2020). The (9S,10E,12Z)-9-hydroperoxy-10,12-octadecadienoic acid (9S-HPODE) stimulates the expression of mycotoxin genes in fungi, while the (9Z,11E,13S)-13-hydroperoxyoctadeca-9,11-dienoic acid (13S-HPODE) does the opposite (Gessler et al. 2017). The 9-hydroxy-10E,12Z-octadecadienoic acid (9-HODE) and the 9-hydroperoxy-10E,12E-octadecadienoic acid (9-HpODE) promote cAMP production via G-protein. This signaling pathway affects germination, sporulation and mycotoxin synthesis. In contrast, the Coriolic acid (13-HODE) and the (9Z,11E)-13-hydroperoxyoctadeca-9,11-dienoic acid (13-HpODE) suppress the production of mycotoxins in *Aspergillus* (Beccaccioli et al. 2019). The mushroom *Agaricus bisporum* is capable of producing 8-HODE and (8R,11S)-dihydroxy-(9Z,12Z)-octadecadienoic acid (8.11-diHODE) from linoleic acid (Wadman et al. 2005).

The presence of oxylipins in the ethanolic extract of *F. rugosa* occurs for environmental reasons, since they are metabolites involved in the regulation of fungal development and communication between the fungus and the host to exploit plant resources and to meet biological needs of fungi (Tsitsigiannis and Keller 2007; Ludovici et al. 2014; Deboever et al. 2019). However, more studies are needed to find the exact role of oxylipins in the ecological relationships of *F. rugosa.*

As previously mentioned, the genus *Ganoderma* belongs to the sister clade of *F. rugosa.* In the studies by Gurovic et al. (2018), the presence of fatty acids in *G. lucidum* caused weak, indirect damaging effects on the prokaryotic cell, which was attributed to an indirect damage of the DNA. Thus, fatty acids should be considered as active components of *G. lucidum*. It is possible that oxylipins contribute to the cytotoxicity of *F rugosa.* More studies are needed to test this hypothesis.

In this study, we evaluated in vitro cell culture models for toxicity screening. HepG-2 is a model for representing liver exposure (Sahu et al. 2014) and glial cell line C6 is a model for representing normal brain astrocytes (Ren et al. 2019). In addition, L929 and Vero have no tumor origin. All lineages have served to mimic healthy cells. The ethanolic extract used here showed weak cytotoxic activity. The standard recommended by the National Cancer Institute is an IC_50_ ≤ 20 μg/ml (Srisawat et al. 2013). The strongest inhibition occurred in HepG-2 (IC_50_ 138.5) and Vero (IC_50_ 119.5) cells. Interestingly, the IC_100_ of the ethanolic extract from *G. lucidum* was 30 μg/mL (Mohan et al. 2016), or an IC_50_ of 31.2 μg/mL, when tested in HepG2 cells. However, it was not cytotoxic to Vero cells (Fathima and Reenaa 2016). The weak cytotoxicity in *F. rugosa* may be explained by the difference in the extract constitution when compared to *G. lucidum*.

Preclinical and clinical studies have demonstrated that *G. lucidum* not only has anti-proliferative effects (Oppatova et al. 2019; Li et al. 2019), but also acts as an anti-influenza (Zhu et al. 2017), neuroprotective (Ren et al. 2019; Lai et al. 2019), anti-aging (Cuong et al. 2019), anti-obesity (Diling et al. 2020) and anti-inflammatory (Su et al. 2020) agent. Thus, we suggest the screening of other biological activities for *F. rugosa* as a future research direction, since we already have the guidelines on cytotoxicity and possible compounds.

## Conclusion

Specimens of the *F. rugosa* mushroom are found in southern Brazil and the identification was confirmed through phylogenetic analysis of the ITS region and 28S gene sequences. The ethanolic extract of *F. rugosa* has weak cytotoxic activity. It may have some cytotoxic constituents. Oxylipins were detected in this extract via LC–MS.

## Supplementary Information

**Additional file 1.** Taxa, vouchers, origin and Genbank accession numbers used in the molecular analysis.

## Data Availability

Accession numbers used in the molecular analysis: GenBank ITS sequences in: https://www.ncbi.nlm.nih.gov/nuccore/MN985326, https://www.ncbi.nlm.nih.gov/nuccore/MN985327. GenBank 28S sequences in: https://www.ncbi.nlm.nih.gov/nuccore/MN985507, https://www.ncbi.nlm.nih.gov/nuccore/MN985508. The final data matrix has been deposited in TreeBASE: http://purl.org/phylo/treebase/phylows/study/TB2:S29283?x-accesscode=335fda807b9242d99ae49933a9a35834&format=html.

## Abbreviations

13-HODE: Coriolic acid
12(13)-EpOME: Vernolic acid
13-Hotre: 13-OH-9Z,11E,15Z-Octadecatrienoic acid
9-KODE: (10E,12Z)-9-Oxooctadeca-10,12-dienoic acid
9, 12, 13-TODEA: 9,12,13-Trihydroxy-10-octadecenoic acid
9,10,13 TriHOME: 9,10,13-Trihydroxy-11-octadecenoic acid
8-HODE: 8-Hydroxylinoleic acid
5.8-diHODE: (5S,8R)-Dihydroxy-octadecadienoic acid
9S-HPODE: (9S,10E,12Z)-9-Hydroperoxy-10,12-octadecadienoic acid
13S-HPODE: (9Z,11E,13S)-13-Hydroperoxyoctadeca-9,11-dienoic acid
9-HODE: 9-Hydroxy-10E,12Z-octadecadienoic acid
9-HpODE: 9-Hydroperoxy-10E,12E-octadecadienoic acid
13-HpODE: (9Z,11E)-13-Hydroperoxyoctadeca-9,11-dienoic acid
8.11-diHODE: (8R,11S)-Dihydroxy-(9Z,12Z)-octadecadienoic acids
