# Supplementary material for: First report of chemical composition and cytotoxicity evaluation of Foraminispora rugosa basidiomata from Brazil
Source: Bot Stud. 2022 Nov 26;63:33. doi: 10.1186/s40529-022-00363-8 (PMC9701286; doi:10.1186/s40529-022-00363-8)
Supplement: Supplementary file 1 — Additional file 1. Taxa, vouchers, origin and Genbank accession numbers used in the molecular analysis. [file 40529_2022_363_MOESM1_ESM.doc]

Botanical Studies

**Supplementary Material A**

First Report on Chemical Composition and Cytotoxicy Evaluation of *Foraminispora rugosa* basidiomata from Brazil

# Garcia V. O.^1,*^, Fronza M.^2^, Von Borowski R.^3^, Alves-Silva G.^1^, Zimmer A. R.^3^, Ruaro T.^3^, Gnoatto S. C. B.^3^, Dallegrave A.^4^ , Silveira R. M. B.^1^

^1^Federal University of Rio Grande do Sul, Institute of Biosciences, Av. Bento Gonçalves 9500, Porto Alegre 91501-970, Rio Grande do Sul, Brazil.

^2^University of Vila Velha, Avenida Comissário José Dantas de Melo, 21 - Boa Vista II, 29102-920, Espírito Santo, Brazil.

^3^Federal University of Rio Grande do Sul, Faculty of Pharmaceutical Sciences, Av. Ipiranga 2752, Porto Alegre 90610-000, Rio Grande do Sul, Brazil.

^4^Federal University of Rio Grande do Sul, Institute of Chemistry, Av. Bento Gonçalves 9500, Porto Alegre 91501-970, Rio Grande do Sul, Brazil.

* Corresponding author. Viviane de Oliveira Garcia. Av. Roraima, 1000. Prédio 21, sala 5231. CEP: 97105-900. Tel.: +55 55 3220.8627. E-mail: voggarcia@yahoo.com.br

Taxa, vouchers, origin and Genbank accession numbers used in the molecular analysis.

| Taxa | Voucher | Origin | ITS | LSU |
| --- | --- | --- | --- | --- |
| 1. *aurantiacum* | FLOR52205 | Brazil | KR816510 | KU315205 |
| *A. aurantiacum* | DHCR540HUEFS | Brazil | KR816511 | MF409953 |
| *A. austrosinense* | CUI13618 | China | KR816512 | KU219996 |
| *A. calcitum* | FLOR50931 | Brazil | KR816513 | KU315207 |
| *A. concentricum* | CUI12648 | China | KR816514 | KU219998 |
| *A. concentricum* | CUI12644 | China | KR816515 | KU219997 |
| *A. elegantissimum* | URM82789 | Brazil | KR816516 | KT006617 |
| *A. elegantissimum* | URM82787 | Brazil | KR816517 | KT006616 |
| *A. intermedium* | FLOR52248 | Brazil | KR816518 | KU315209 |
| *A. intermedium* | FLOR52246 | Brazil | KR816519 | KU315208 |
| *A. omphalodes* | DHCR499/501HUEFS | Brazil | KR816520 | MF409951 |
| *A. omphalodes* | DHCR500HUEFS | Brazil | KR816521 | MF409952 |
| *A. perplexum* | DAI10811 | China | KR816522 | KU220002 |
| *A. perplexum* | CUI6496 | China | KR816523 | KU220001 |
| *A. rugosum* | CUI8795 | China | KR816524 | KU220004 |
| *A. rugosum* | DAI13716 | China | KR816525 | KU220007 |
| *A. schomburgkii* | FLOR52177 | Brazil | KR816526 | KU315215 |
| *A. schomburgkii* | URM83228 | Brazil | KR816527 | KT006621 |
| *A. yunnanense* | CUI7974 | China | KR816528 | KU220013 |
| *F. biseptata* | FLOR50932 | Brazil | KR816529 | KU315206 |
| *F. rugosa* | FLOR52191 | Brazil | KR816530 | KU315216 |
| *F. rugosa* | DHCR554HUEFS | Brazil | KR816531 | MF409954 |
| *F. rugosa* | DHCR560HUEFS | Brazil | KR816532 | MF409955 |
| *G. applanatum* | DAI12483 | China | KR816533 | KF495009 |
| *G. applanatum* | WEI5787 | China | KR816534 | KF495011 |
| *G. australe* | DHCR411HUEFS | Brazil | KR816535 | MF436672 |
| *G. australe* | DHCR417HUEFS | Brazil | KR816536 | MF436673 |
| *G. australe* | FLOR52289 | Brazil | KR816537 | KU315217 |
| *G. australe* | URM83325 | Brazil | KR816538 | JX310802 |
| *G. lucidum* | WD565 | China | KR816539 | AB368068 |
| *G. mbrekobenum* | UMN7-3 | Ghana | KR816540 | KX000897 |
| *G. mbrekobenum* | UMN7-4 | Ghana | KR816541 | KX000899 |
| *G. orbiforme* | URM83334 | Brazil | KR816542 | JX310828 |
| *G. orbiforme* | URM83332 | Brazil | KR816543 | JX310827 |
| *G. parvulum* | URM2948 | Brazil | KR816544 | JX310835 |
| *G. parvulum* | URM83339 | Brazil | KR816545 | JX310831 |
| *G. sinense* | WEI5327 | China | KR816546 | KF495008 |
| *G. sp* | GD026HUEFS | Indonesia | KR816547 | MF436671 |
| *G. tropicum* | KUMCC18-0046B | Thailand | KR816548 | MH823540 |
| *G. tropicum* | HE1232 | Brazil | KR816549 | KF495010 |
| *G. tsugae* | AFTOLID771 | China | KR816550 | AY684163 |
| *G. weberianum* | CBS219-36 | Philippines | KR816552 | MH867289 |
| *Perenniporia medula- panis* | CBS457.48 | Canada | MH856433 | - |
| *Perenniporia medula- panis* | MUCL43250 | China | KR816553 | FJ393875 |
| *T. colossus* | URM83330 | Brazil | KR816554 | JX310811 |
| **GAS1084** | ICN200399 | Brazil | MN985327 | MN985508 |
| **VOG127** | ICN200398 | Brazil | MN985326 | MN985507 |

* The accession numbers in bold-face indicate the newly generated sequences for this study.
